# Prognostic Usefulness of Motor Unit Number Index (MUNIX) in Patients Newly Diagnosed with Amyotrophic Lateral Sclerosis

**DOI:** 10.3390/jcm12155036

**Published:** 2023-07-31

**Authors:** Barbara Risi, Stefano Cotti Piccinelli, Stefano Gazzina, Beatrice Labella, Filomena Caria, Simona Damioli, Loris Poli, Alessandro Padovani, Massimiliano Filosto

**Affiliations:** 1 Department of Clinical and Experimental Sciences, University of Brescia, 25121 Brescia, Italy; barbara.risi@centrocliniconemo.it (B.R.); stefano.cottipiccinelli@centrocliniconemo.it (S.C.P.); beatrice.labella93@gmail.com (B.L.); alessandro.padovani@unibs.it (A.P.); 2 Unit of Neurology, ASST Spedali Civili, 25123 Brescia, Italy; loris.poli@asst-spedalicivili.it; 3 NeMO-Brescia Clinical Center for Neuromuscular Diseases, 25064 Gussago, Italy; filomena.caria@centrocliniconemo.it (F.C.); simona.damioli@centrocliniconemo.it (S.D.); 4 Unit of Neurophysiology, ASST Spedali Civili, 25123 Brescia, Italy; stefano.gazzina@asst-spedalicivili.it

**Keywords:** amyotrophic lateral sclerosis (ALS), motor unit number index (MUNIX), disease progression rate (DPR), neurophysiology, prognosis

## Abstract

The MUNIX technique allows us to estimate the number and size of surviving motor units (MUs). Previous studies on ALS found correlations between MUNIX and several clinical measures, but its potential role as a predictor of disease progression rate (DPR) has not been thoroughly evaluated to date. We aimed to investigate MUNIX’s ability to predict DPR at a six-month follow up. Methods: 24 ALS patients with short disease duration (<24 months from symptoms’ onset) were enrolled and divided according to their baseline DPR into two groups (normal [DPR-N] and fast [DPR-F] progressors). MUNIX values were obtained from five muscles (TA, APB, ADM, FDI, Trapezius) and averaged for each subject. Results: MUNIX was found to predict DPR at follow up in a multivariable linear regression model; namely, patients with lower MUNIX values were at risk of showing greater DPR scores at follow up. The result was replicated in a simple logistic regression analysis, with the dichotomic category “MUNIX-Low” as the independent variable and the outcome “DPR-F” as the dependent variable. Conclusions: our results pave the way for the use of the MUNIX method as a prognostic tool in early ALS, enabling patients’ stratification according to their rates of future decline.

## 1. Introduction

Amyotrophic lateral sclerosis (ALS) is a fatal neurodegenerative disease that affects both the upper (UMN) and lower (LMN) motor neurons [1]. Neurophysiological techniques are widely used to explore disease-associated UMN and LMN changes, and the role of conventional electroneuromyography (ENG-EMG) has long been recognized in several diagnostic criteria, including the most recent Gold Coast criteria [2].

ALS is dominated by a well-known clinical heterogeneity in terms of both involved systems and evolution over time, and there is an urgent, unmet need for biomarkers to enable better stratification of patients [1]. 

Several tools have been developed to measure ALS burden and progression over time, but none of them appear to be fully satisfactory and capable of simultaneously capturing all facets of the disease. Indeed, the aforementioned clinical variability, relating to the site of onset, the pattern of progression through different body regions and the prevalence of upper rather than lower motor neuron involvement, requires different and specific outcome measures [3]. The ALS Functional Rating Scale Revised (ALSFRS-R) [4] is one of the most widely used instruments for evaluating patients’ functional status and is the gold standard for primary efficacy outcomes in clinical trials. In the work by Kaufmann et al. [5], the authors found that baseline ALSFRS-R is a strong predictor of death or tracheostomy at 2-year follow up, regardless of forced vital capacity and after adjustment for age, sex and symptoms’ duration. Further data from a Japanese cohort [6] showed that the disease progression rate (DPR) at baseline [calculated as “difference in ALSFRS-R (48 - observed score)/duration from symptoms’ onset”], appears more closely associated with survival, providing an additional predictive index beyond ALSFRS-R alone. In addition, some limitations of ALSFRS-R are to be mentioned, among which the inherent subjectivity, the relative insensitivity to progression over short periods of time and the susceptibility to patient’s mood. 

Hand-held dynamometry (HHD) enables the testing of limb strength, but again it is largely operator dependent, needs up to three tests to ensure data consistency, lacks reliability in the extremes of the strength spectrum (i.e., in very strong patients, operator’s own strength risks to be measured too) and is affected by mood, effort and cognitive status [7].

Bulbar weakness assessment could be afforded through specific tests like the Iowa Oral Performance Instrument measurements of tongue, lip and cheek strength and the Sydney Swallow Questionnaire [8,9].

Forced vital capacity (FVC) is a good indicator of respiratory decline and can rely on well-developed normative data, but its performance is prevented in later disease stages by bulbar and oral weakness [10]. Instead, sniff nasal inspiratory pressure (SNIP) is recommended because it balances reliability in most ALS patients and has greater sensitivity to changes in respiratory muscle strength [11]. 

Several neurophysiological and imaging techniques have been exploited to investigate corticospinal tract and cortical network disruption, as well as enhanced hyperexcitability, namely transcranial magnetic stimulation (TMS) [12,13] and magnetic resonance imaging (MRI) with diffusion tensor imaging (DTI) and quantitative susceptibility mapping [14]. Nevertheless, their use is cost and time consuming, not so commonly disseminated in clinical practice, and regarding the latter, limited to patients able to remain supine; a posture often not achievable in the later disease phases. 

Much progress has been made in the field of so-called ‘wet’ biomarkers, including some non-specific ones (creatine kinase, uric acid and serum cholesterol, which do correlate with survival) [15,16,17] and others, reflecting the extent of axonal damage, such as neurofilaments (NFs); plasma NF-L subunit (neurofilament light chain) has been shown to identify phenoconversion in clinically asymptomatic mutant SOD1 carriers and to predict future ALSFRS-R slope and survival [18,19]. 

ENG has limited yield in quantifying axonal loss because compound muscle action potential amplitude (CMAP) remains above the lower limit of normal even with a loss of >50% of axons, and needle EMG is not very suitable for serial studies because it is generally uncomfortable [20].

A quantitative study of surviving motor units was made possible by the advent of motor unit number estimation techniques, which all rely on the ratio between the supramaximal CMAP amplitude and the average surface-recorded motor unit potential (SMUP) amplitude, and to which the motor unit number index (MUNIX) belongs. Among the MUNE methods’ pitfalls to be counted, there is the need for substantial operator training. Nevertheless, they show good inter-rater and test–retest reliability. MUNIX has been applied to small, distal limb muscles, including the abductor pollicis brevis (APB), abductor digiti minimi (ADM), extensor digitorum brevis (EDB) and abductor hallucis (HA), as well as to larger muscles such as the biceps brachii (BB), tibialis anterior (TA) and trapezius [21,22,23,24]. The motor unit size index (MUSIX) is another electrophysiological parameter, derived from MUNIX. It mirrors the size of motor units (MUs) and its increase is related to progressive motor units ’enlargement during axonal loss over time and it is a quantitative outcome measure of functional reinnervation [21].

MUNIX has been studied as a diagnostic and monitoring marker in ALS. It is extremely sensitive to subtle changes in the number of functioning motor units, demonstrating LMN loss in pre-symptomatic limbs of ALS patients [25,26,27]. It correlates with ALSFRS-R, CMAP amplitude and spirometry measures such as slow vital capacity [28,29,30]. Moreover, in longitudinal studies comparing different clinical measures, MUNIX values showed faster rates of decrease than ALSFRS-R, CMAP amplitude or manual muscle testing (Medical Research Council [MRC] scale), revealing major sensitivity to disease progression [31,32,33,34]. MUSIX’s increase has been associated with a relative preservation of muscle strength despite a significant loss of motor units, thus measuring a clinically relevant reinnervation effect [21].

On the other hand, MUNIX has never been explored as a prognostic tool, and it is not known whether MUNIX values can predict disease aggressiveness over time, measured as disease progression rate (DPR). Defining homogeneous populations eligible for clinical trials is an important and crucial challenge for clinicians, as heterogeneity in ALS aggressiveness may reflect different disease mechanisms and thus different susceptibility to treatment [35]. Furthermore, it would be useful to stratify patients at the first visit according to the likely future trend, to ensure better disease management in clinical practice (e.g., NIV and gastrostomy timing).

The aim of the present study was to investigate whether MUNIX parameters at baseline could be useful in predicting disease progression rate (DPR) after six months.

## 2. Materials and Methods

### 2.1. Patients

Consecutive ALS patients were screened at two Brescia University Centers, the ASST Spedali Civili Neurology Unit and the NeMO-Brescia Clinical Center for Neuromuscular Diseases.

We included patients selected by the following inclusion criteria: clinical diagnosis of ALS (definite, probable, laboratory-supported probable, or possible) according to the El-Escorial revised criteria [36], age over 18 years old, disease duration (from symptoms’ onset) <24 months and ability to provide informed consent to the study. This study was approved by the local ethics committee (NP 5470, NP 5471) and performed in conformity with the Helsinki Declaration. Informed consent was obtained from all participants.

A sample of electrophysiological reference values from 22 healthy subjects (HS) was obtained from an internal machine dataset previously acquired for calibration purposes.

### 2.2. Clinical and Laboratory Evaluation

At enrolment, demographic and clinical data, including ALS history (site of onset, date of symptoms’ onset, date of diagnosis, genotype), were recorded in an appropriate database.

The ALSFRS-R questionnaire was administered to all patients at enrolment and at six-month follow up.

Patients were divided into two groups according to their DPR, calculated as “48-ALSFRS-R/disease duration in months”: normal progressors [DPR-N] if DPR was <1.1 points/month; fast progressors [DPR-F] if DPR was ≥1.1 points/month. These cut offs were chosen because they are already widely used in the literature [37]. 

Respiratory function (forced vital capacity, FVC), cerebrospinal fluid (CSF) and brain MRI-DTI findings were also collected. 

At baseline and at each follow up, the MRC scale was applied to the muscles tested with MUNIX with a score ranging from 0 (no contraction) to 5 (normal strength); a score of 4.5 was assigned to patients with strength rated 4+/5 and 3.5 to patients with strength rated 4−/5 [38]. Contextually, strength was measured in the same muscles by using a hand-held dynamometer (MicroFET2 HHD) [39]. Testing occurred just after the MUNIX examination with the patient seated in a hardback chair, and two different pads (concave and finger pad) were used according to the muscles tested to better fit their contour. Patients were encouraged to elicit as much force as possible against the HHD, matching the operator’s resistance at isometric contraction for at least 4 s. For each muscle, two readings were obtained and subsequently averaged; in case of >15% of variability [calculated as (Max value − Min value)/(Max value) × 100)], a third trial was performed. 

### 2.3. MUNIX Procedure

MUNIX is a two-step procedure [21,40,41,42]. The first one is the recording of the supramaximal CMAP (baseline to negative peak), obtainable using standard motor nerve conduction techniques. After positioning the stimulator, current intensity is gradually increased until the CMAP plateau, followed by further increase by additional 20% to ensure supramaximal stimulation. Once supramaximal stimulation is achieved, appropriate replacement of the recording electrode is performed to maximize CMAP amplitude. CMAP amplitude greater than 0.5 mV is required for the subsequent computation. Disposable, self-adhesive surface ground and 15 mm disk recording electrodes were used. The second step is to record “surface electromyographic interference pattern” (SIP): at least 20 epochs of 500 msec duration, during increasing voluntary contraction at different force levels, ranging from slight to maximum (with special emphasis on low and mid-range of force). The contraction is isometric, with the investigator providing resistance. The high-pass filter was set at 2–3 Hz, the low-pass filter at 3 kHz. SIPs that did not fulfil some quality criteria (such as quality index below 1.0, ICMUC > 100 and SIP area < 20 mV/ms) were automatically rejected. SIP recordings with artefacts, baseline shift, tremor or obvious change of force level within the single epoch were avoided. Data were collected using Neuro-MEP.NET EMG software (version 4.2.5.9, Neurosoft LLC, Ivanovo, Russia). Subjects were seated in a comfortable chair with a table nearby to support their hands during the measurements; attention was paid to limb position and recording electrode replacement to ensure consistency between repeated measurements and maximize CMAP amplitude, respectively. MUNIX was measured on the less-affected side. The choice of limiting the analysis to one upper and one lower limb was taken for two reasons: first, because we wished to avoid a time-consuming examination, in view of its possible application in longitudinal designs, and second, in a few cases the choice was almost forced due to marked contralateral limb weakness, such that the examination was not possible, as minimal patient cooperation is required to effect the contraction. The choice of measuring just the limbs of one side, namely the less affected one, has already been carried out by some study groups [22,25,27,28,43]. 

Taking this issue into account, due to the partly advanced muscle wasting in some patients, MUNIX examination was not always feasible in all the muscles even considering the less-affected side. Therefore, to avoid selection bias and reduce the flawed deviations towards higher median values, we set each measurement without such a value as follows: MUNIX = 2, MUSIX = 250 and CMAP = 0.5 mV. These cut-offs have been recently proposed in two works [43,44]. As a result, there were no missing values in the data processed for statistical analyses.

The following five muscles were tested: 1. abductor pollicis brevis (APB); 2. abductor digiti minimi of the hand (ADM); 3. first dorsal interosseus of the hand (FDI); 4. tibialis anterior (TA) and 5. trapezius (Trap). The electrode placement for individual muscles was standardized according to guidelines [23,42].

To allow comparisons between groups, the average per subject of the MUNIX and MUSIX values derived from the five muscles (henceforth referred to as “MUNIXmean” and “MUSIXmean”) was obtained.

### 2.4. Statistical Analysis

Statistical analysis was performed with SPSS 26 (IBM, Armonk, NY, USA) software. Data were presented as median (25th–75th percentile) and significance was set at *p* < 0.05 for all analyses. Comparisons of baseline clinical characteristics between DPR-N and DPR-F groups were evaluated using the Mann–Whitney U test for continuous variables and chi-square for dichotomous variables. Fisher’s exact test was used when at least one expected frequency in a fourfold table was less than five. 

A multivariable linear regression analysis with backward elimination at *p* > 0.1 was used to assess the best predictors of disease progression at follow up.

A simple logistic regression analysis was used to predict the outcome “DPR-F” using the category “MUNIX-Low” as independent variable; “MUNIX-Low” was obtained by dichotomizing MUNIXmean values based on the sample median (cut-off, i.e., 114).

Spearman correlation analyses between MUNIX parameters and clinical variables were adjusted for the effects of age, sex and disease duration.

## 3. Results

### 3.1. Clinical and Electrophysiological Data across DPR Groups at Baseline

**Figure 1:**
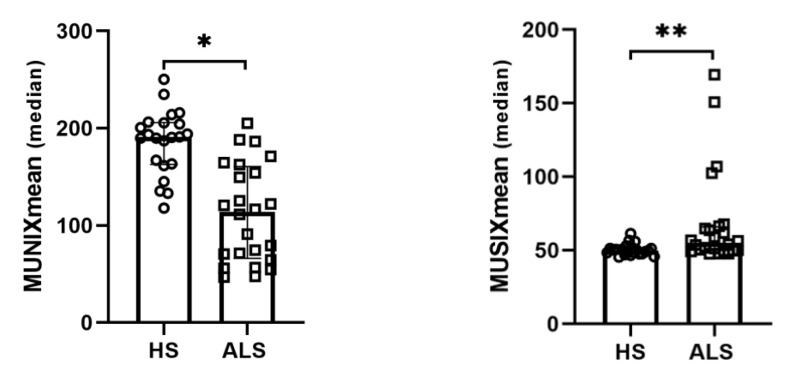
Scatter plots with bar showing MUNIXmean and MUSIXmean in HS and ALS. Data are shown as median with interquartile range. Abbreviations: ALS = Amyotrophic lateral sclerosis; HS = healthy subjects; MUNIXmean = the mean of motor unit number index; MUSIXmean = the mean of motor unit size index. * *p* < 0.001. ** *p* = 0.001.

We screened 24 ALS patients. Table 1 shows demographic features for the ALS and HS groups. ALS patients showed a lower MUNIXmean (*p* < 0.001) and a higher MUSIXmean (*p* = 0.001) than HS (Figure 1) in age- and sex-adjusted ANCOVA. 

Median MUNIX, MUSIX, CMAP and HHD values for each muscle in HS and ALS groups are reported in Table S1. Single MUNIX and MUSIX values for each muscle in ALS subjects are reported in Table S2.

Dividing patients according to their baseline DPR, 16 patients were classified as DPR-N and 8 as DPR-F (Table 2). The DPR-F group showed lower ALSFRS-R (*p* < 0.001), ALSFRS-R bulbar subscore (*p* = 0.045) and FVC percentage (*p* = 0.010) values and higher frequency of NIV use (*p* = 0.002), as expected.

Regarding genotype characterization, molecular data were available for 20 patients. In *n* = 19, a large panel of genes potentially associated with ALS was analyzed by NGS method, whereas in *n* = 1 the analysis was limited to C9orf72, SOD1, FUS, TARDBP and VCP genes. Genetic analysis revealed: no mutation in *n* = 11/20, variants of uncertain significance (VUS) in one or more genes in *n* = 3/20, likely pathogenic variants (in DHTKD1, LRP10 and FGGY genes) in *n* = 3/20 and known pathogenic variants (in SQSTM1, C9orf72 and TARDBP genes) in *n* = 3/20. Focusing on the latter group, the patient mutated in SQSTM1 was a rapid progressor at baseline, while the other two showed normal progression and remained like this at the six-month follow up.

No patients at baseline had a gastrostomy or tracheostomy.

### 3.2. MUNIX Parameters as Predictors of DPR in Newly Diagnosed ALS Patients 

During follow up, five ALS patients moved from the DPR-N to the DPR-F group. Table 3 shows clinical features at follow up. At follow up, three more patients required NIV, three required gastrostomy and one required tracheostomy.

MUNIXmean, MUSIXmean, and other clinical variables (gender, age, bulbar site of onset, baseline ALSFRS-R, baseline DPR, use of riluzole) were analyzed by a multivariable linear regression, to evaluate the best predictors of disease progression rate at six months [DPR_T6_] (Table 4).

MUNIXmean was revealed to be a significant predictor of DPR_T6_ (β = −0.228, *p* = 0.033), along with baseline DPR (β = 1.053, *p* < 0.001). 

In addition, we transformed MUNIXmean values into a dichotomous variable, using the sample median of 114 as the cut off, and found that 12 patients had mean values below it, henceforth referred to as “MUNIX-Low” group. The MUNIX-Low category revealed to be a risk factor for fast progression at follow up in a logistic regression model (Exp[B] = 6.000, *p* = 0.048) (Table 5). 

**Figure 2:**
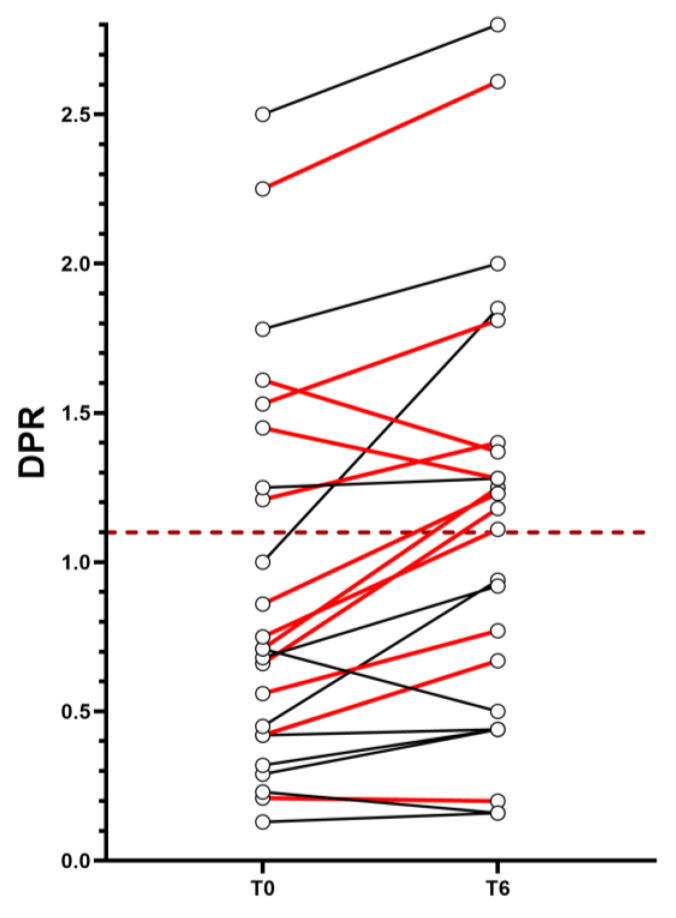
DPR values for single subject at T0 and T6. The graph shows the trend of DPR per subject from T0 (left) to T6 (right). The dashed horizontal line identifies the boundary between DPR-N (below it) and DPR-F (above it). The red connecting lines between the two time points identify subjects belonging to the MUNIX-Low group, while the black connecting lines identify subjects not belonging to the MUNIX-Low group. Abbreviations: DPR = disease progression rate.

Finally, of the five patients who transitioned from the DPR-N to the DPR-F group during the six-month follow up, four (80%) belonged to the MUNIX-Low category (Figure 2). At follow up, two patients out of these four required gastrostomy placement and one of them required NIV use.

### 3.3. Correlation with Clinical Variables

MUNIXmean exhibited a positive correlation with baseline ALSFRS-R (r = 0.576; *p* = 0.006). The two parameters, MUNIXmean and MUSIXmean, showed a negative correlation with each other (r = −0.605, *p* = 0.004). No correlation was revealed between MUNIX parameters and baseline FVC (%), CSF pTau/Tau ratio or BMI values. 

Correlations between MUNIX parameters and CMAP, MRC, and HHD values within each muscle are shown in the Supplementary Materials.

## 4. Discussion

This study evaluated the MUNIX technique in a cohort of 24 ALS patients with a short disease duration. The diagnostic delay in our cohort was at the lower limits of the range reported in the literature (i.e., 9.1–27 months) [45].

The main finding of our study is that, in newly diagnosed ALS patients, MUNIXmean values could predict the DPR at six months. 

Patients were classified at baseline as normal and fast progressors, according to their baseline DPR. Significantly different clinical features among the DPR groups were the ALSFRS-R and the respiratory function (FVC %) values, which appeared lower in the DPR-F group, a sign of more advanced denervation.

Previous studies found a correlation between MUNIX values and ALSFRS-R scores and demonstrated that they both decline over time, with MUNIX declining in a more pronounced way than ALSFRS-R; however, none of them investigated MUNIX as a predictor of future DPR, a more informative measure than raw ALSFRS-R [30,46].

We decided to enroll patients with a short disease history for two reasons: firstly, the patients more likely to be eligible for disease-modifying pharmacological interventions are the newly diagnosed, and our results are therefore applicable to this setting; secondly, disease aggressiveness may not yet be defined in the early stages, as shown by the case of four patients in our cohort who went from “normal” to “fast progression” within six months. This dynamic behavior of the DPR reflects the recently proposed model of ALS progression as a sigmoidal, rather than linear, trajectory [47]. According to this theory, each patient goes through three recognized phases [relative stability (phase I), early progression (phase II), late progression and stability (phases III–IV)], and the slope of the curve defines the speed of progression. Thus, a patient with an apparently slow progression in the first few months (ideally, in phase I) might accelerate his speed of progression to such an extent that he becomes a “fast progressor” in the following phases.

In our study, the rate of disease progression at six months was predicted by MUNIXmean in a multivariable linear regression model; precisely, patients with lower values were more likely to show greater DPR scores after six months. Applying a dichotomization of MUNIXmean, patients with values below the median (“MUNIX-Low”) were at risk to be “fast progressors” at six months, according to a logistic regression analysis. We tried to apply the proposed model at the single-subject level. Seven out of the twelve patients belonging to the MUNIX-Low category at baseline were in the DPR-N group and were therefore potentially at risk of evolving to DPR-F over six months, according to the model. At follow up, four (57.2%) of them actually shifted from normal to fast progression, while three (42.8%) remained in the DPR-N group. However, the small sample size must be taken into account in the interpretation of this result, and we believe that a larger cohort is needed to identify more precise cut-offs and validate the proposed predictive model.

In summary, our preliminary findings showed that the presence of few MUs in the first months after the onset of symptoms reflects a potentially rapid disease. The reason why MUSIX did not emerge as a significant predictor in the model could be that in the early stages the neuropathological picture is dominated by denervation, with re-innervation not yet evident and unable to differentiate fast from normal progressors.

As is well recognized, the neuropathological process of ALS starts long before symptoms onset. Both animal (with mutant SOD1 transgenic mice) and human autopsy studies, revealed that early motor neuron loss precedes the manifest disease, as evident from the anterior roots’ neuronal depletion and the rise of neurofilament (NFs) levels in the pre-symptomatic phase [18,48,49]. Our results introduce the idea of a quantifiable motor neuron reserve, which allows the clinical course of the disease to be delineated on the basis of the biological stage. The use of MUNIX could have an impact on trial design, helping patients’ stratification according to the likelihood of progression in the short term. Assuming that the heterogeneity of the aggressiveness of ALS may reflect different pathological mechanisms leading to an unpredictable and possibly divergent treatment effect, recent trials have targeted selected populations of “normal progressors” to ensure the homogeneity of the sample [20]. In this perspective, it would be useful to distinguish more precisely between normal and fast-progressing subjects in the early stages of the disease. 

Collaterally, our study demonstrates that MUNIX values at each muscle level correlate well with the strength measured by the MRC scale and HHD, being therefore a reliable parameter of clinical involvement.

Possible future approaches of the MUNIX method include its integration within multi-biomarkers composed batteries, possibly in association with specific measurement scales for the upper motor neuron involvement. We also believe it could be usefully integrated within protocols of artificial intelligence, in particular to define disease progression patterns from one body region to another.

This study has some limitations: first, as already stated, the relatively small number of the study sample, limited by the choice of including only patients with a few months of disease history; second, the follow up limited to six months, even if we think this is a reasonable time window to track the evolution of the disease, especially within a clinical trial; third, the method may not adequately capture the progression in patients with prevalent involvement of the UMN and this should be considered in the selection of patients to be examined.

## 5. Conclusions

Our preliminary findings suggest the MUNIX exam as a prognostic tool in newly diagnosed ALS patients, as well as the need to confirm our results in larger cohorts, in order to identify reliable cut-off values and to contribute to patients’ clustering within clinical settings and trials.

## Figures and Tables

**Table 1:** Demographic features and MUNIX parameters in HS and ALS.

	HS (*n* = 22)	ALS (*n* = 24)	*p*-Value
**Age, years**	53.2 (30.6–67.8)	65.2 (61.7–73.6)	**0.007**
**Sex M, *n* (%)**	8 (36.4)	16 (66.7)	**0.040**
**Dis. duration, months**	-	13 (11.2–19)	
**MUNIXmean**	190.8 (163–205.8)	114 (66–160.5)	<0.001 *
**MUSIXmean**	50 (47.7–51.4)	55.3 (49.9–66)	0.001 **

Parameters are given as median (25°–75° percentile). Abbreviations: ALS = Amyotrophic lateral sclerosis; HS = healthy subjects; MUNIXmean = the mean of motor unit number index; MUSIXmean = the mean of motor unit size index. * *p* < 0.001 and ** *p* = 0.001 age- and sex-adjusted ANCOVA.

**Table 2:** Demographic and baseline clinical features across DPR groups.

		Total (*n* = 24)	DPR-N (*n* = 16, 66.7%)	DPR-F (*n* = 8, 33.3%)	*p*-Value
**Age, years**		65.2 (61.7–73.6)	64.5 (61.7–72.5)	66.8 (57.4–75)	0.697
**Sex M, *n* (%)**		16 (66.7)	12 (75)	4 (50)	0.363
**ALS duration, months**		13 (11.2–19)	14 (12–19)	12 (12–12)	0.787
**Diagn. delay, months**		9 (7–12.7)	12 (7–13)	8 (8–8)	0.976
Genetic, *n* (%) *		3 (12.5)	2 (12.5)	1 (12.5)	1.000
**Bulbar onset, *n* (%)**		8 (33.3)	4 (25)	4 (50)	0.363
**El Escorial, *n* (%)**	**Def**	2 (8.3)	2 (12.5)	0	0.483
	**Prob**	12 (50)	8 (50)	4 (50)	
	**Lab-Prob**	6 (25)	3 (18.8)	3 (37.5)	
	**Poss**	4 (16.7)	3 (18.8)	1 (12.5)	
**ALSFRS-R**		39 (32.2–43)	42 (39–43)	27 (21–33)	**<0.001**
**ALSFRS-R bulbar**		9.5 (8.2–11.7)	10.5 (9–12)	8.5 (6.2–10.5)	**0.045**
**Riluzole use, *n* (%)**		20 (83.3)	12 (75)	8 (100)	0.262
BMI **		24 (21–27.5)	24 (23.3–26)	30.8 (30–31.6)	0.856
**NIV, *n* (%)**		13 (54.2)	5 (31.3)	8 (100)	**0.002**
CSF ***	**Tau**	258 (220–407.5)	245 (199–315)	334 (258–410)	0.287
	**pTau**	29 (24–44.5)	27 (22–39)	39.5 (29–50)	0.371
	**pTau/Tau**	0.12 (0.10–0.13)	0.12 (0.10–0.13)	0.11 (0.1–0.13)	0.371
↓ DTI-FA, *n* (%) ****		16 (84.2)	12 (85.7)	4 (80)	1.000
FVC % *****		81.5 (53–93.5)	91.5 (75–101.5)	54 (50.5–81)	**0.010**
**MUNIXmean**		114 (66–160.5)	111.4 (74.5–171.1)	89.4 (56.7–122)	0.070
**MUSIXmean**		55.3 (49.9–66)	56.4 (50.4–63.5)	80.07 (58.9–102.5)	0.136

* Available in *n* = 20/24, ** available in *n* = 21/24; *** available in *n* = 13/24; **** available in *n* = 19/24; ***** available in *n* = 20/24. Abbreviations: ALS = Amyotrophic lateral sclerosis; ALSFRS-R = ALS functional rating scale revised; BMI = body mass index; CSF = cerebrospinal fluid; DPR = disease progression rate; DPR-N = normal progressors; DPR-F = fast progressors; DTI-FA = diffusion tensor imaging—fractional anisotropy; FVC = forced vital capacity; MUNIXmean = the mean of motor unit number index; MUSIXmean = the mean of motor unit size index; NIV = non-invasive ventilation; BMI is given as kg/m^2^. Tau and pTau are given as ng/mL.

**Table 3:** Clinical features across DPR groups at follow-up.

	DPR-N (*n* = 11, 45.8%)	DPR-F (*n* = 13, 54.2%)	*p*-Value
Genetic, *n* (%) *	2 (18.2)	1 (7.7)	0.576
**ALSFRS-R**	36.5 (33–42.5)	19 (15–24.2)	**0.003**
**NIV, *n* (%)**	8 (72.7)	8 (61.5)	**0.022**
**Gastrostomy, *n* (%)**	1 (9.1)	2 (15.4)	1.000
**Tracheostomy, *n* (%)**	0	1 (7.7)	1.000

* Available in *n* = 20/24. Abbreviations: ALSFRS-R = ALS functional rating scale revised; DPR = disease progression rate; DPR-N = normal progressors; DPR-F = fast progressors; NIV = non-invasive ventilation.

**Table 4:** Multivariable linear regression model for DPR_T6_, based on demographics and baseline clinical variables: (a) initial model and (b) model after backward elimination.

(a)					
Independent Variables	B	Standard Error	Beta	*t*	*p*-Value
**Constant**	0.558	0.955		0.584	0.568
**MUSIXmean**	−0.005	0.003	−0.226	−1.872	0.081
**MUNIXmean**	−0.003	0.002	−0.235	−2.103	0.053
**Gender**	0.132	0.120	0.088	1.102	0.288
**Age**	−0.005	0.008	−0.076	−0.718	0.484
**Site of onset (bulbar)**	0.024	0.121	0.016	0.200	0.844
**Baseline ALSFRS−R**	0.019	0.011	0.224	1.737	0.103
**Baseline DPR**	1.144	0.119	1.038	9.624	**0.000**
**Riluzole**	−0.184	0.167	−0.097	−1.101	0.288
**(b)**					
**Independent variables**	**B**	**Standard Error**	**Beta**	***t***	***p-*** **Value**
**Constant**	−0.116	0.451		−0.257	0.800
**MUSIXmean**	−0.003	0.002	−0.153	−1.851	0.080
**MUNIXmean**	−0.003	0.001	−0.228	−2.294	**0.033**
**Baseline ALSFRS−R**	0.021	0.010	0.250	2.101	**0.049**
**Baseline DPR**	1.161	0.111	1.053	10.472	**0.000**

Abbreviations: ALSFRS-R = ALS functional rating scale revised; DPR = disease progression rate; MUNIXmean = the mean of motor unit number index; MUSIXmean = the mean of motor unit size index.

**Table 5:** Logistic regression model, with “DPR-F” group as outcome.

Variables	B	S.E.	Wald	gl	Sign.	Exp(B)
**MUNIX-Low**	1.792	0.905	3.918	1	**0.048**	6.000
**Constant**	−0.693	0.612	1.281	1	0.258	0.500

Abbreviations: DPR-F = disease progression rate—fast progressors; MUNIX-Low = motor unit number index, the values below the cut off (median).

## Data Availability

The data that support the findings of this study are available from the corresponding author (M.F.), upon reasonable request.

## Supplementary Materials

The following supporting information can be downloaded at: https://www.mdpi.com/article/10.3390/jcm12155036/s1, Table S1. MUNIX parameters, MRC and HHD values per muscle in HS and ALS. Table S2. Individual MUNIX parameters per muscle in ALS.
