# Supplementary material for: Prognostic Usefulness of Motor Unit Number Index (MUNIX) in Patients Newly Diagnosed with Amyotrophic Lateral Sclerosis
Source: J Clin Med. 2023 Jul 31;12(15):5036. doi: 10.3390/jcm12155036 (PMC10420094; doi:10.3390/jcm12155036)
Supplement: Supplementary file 1 [file jcm-12-05036-s001.zip › jcm-2485148-supplementary.pdf]

SUPPLEMENTARY MATERIAL

Prognostic usefulness of Motor Unit Number Index (MUNIX) in patients newly diagnosed with Amyotrophic Lateral Sclerosis

Barbara Risi MD[1,2,3], Stefano Cotti Piccinelli MD[1,3], Stefano Gazzina MD[4], Filomena Caria MD[3], Simona Damioli MD[3], Beatrice Labella MD[1,2], Loris Poli MD[2], Alessandro Padovani MD, PhD[1,2], Massimiliano Filosto MD, PhD[1,3]*

[1]Department of Clinical and Experimental Sciences, University of Brescia, Brescia, Italy

[2]Unit of Neurology, ASST Spedali Civili, Brescia, Italy

[3]NeMO-Brescia Clinical Center for Neuromuscular Diseases, Brescia, Italy

[4]Unit of Neurophysiology, ASST Spedali Civili, Brescia, Italy

*Corresponding author:

Massimiliano Filosto

Department of Clinical and Experimental Sciences, University of Brescia; NeMO-Brescia Clinical Center for Neuromuscular Diseases

Via Paolo Richiedei 16, 25064 Gussago (BS), Italy

Tel: (+39)030-6729100. E-mail: massimiliano.filosto@unibs.it

Table S1. MUNIX parameters, MRC and HHD values per muscle in HS and ALS.

		HS (n=22)	ALS (n=24)	p-value
**APB**	**MUNIX**	166 (128.2-196.5)	61.3 (42.4-137.5)	**<0.001**
	**MUSIX**	49.2 (45.7-54)	51.5 (47.8-68.7)	0.057
	**CMAP**	7.7 (6.6-9.3)	4.4 (2.5-6.8)	**<0.001**
	**MRC**	-	4.5 (3.6-5)	
	**HHD**	-	4.9 (0-9.6)	
**ADM**	**MUNIX**	142.5 (120.7-197)	81.6 (54.4-159.5)	**0.003**
	**MUSIX**	55.8 (50.3-63.8)	58.8 (53.6-77.9)	0.141
	**CMAP**	9.4 (6.8-10.9)	6 (3.2-8.7)	**0.001**
	**MRC**	-	4.5 (3.6-5)	
	**HHD**	-	0 (0-4.2)	
**FDI#**	**MUNIX**	266 (219-310)	144 (71.4-201.7)	**<0.001**
	**MUSIX**	56.4 (49-63.7)	64.2 (53.174.3)	**0.024**
	**CMAP**	14.4 (12.6-16.8)	8.4 (4.1-11.7)	**<0.001**
	**MRC**	-	4.5 (3.6-5)	
	**HHD**	-	3.5 (0-4.6)	
**TA**	**MUNIX**	133.5 (119-55.5)	86.7 (56-123.5)	**0.001**
	**MUSIX**	47.6 (43.1-50.9)	51.9 (49-56.6)	**0.002**
	**CMAP**	6.2 (5.7-7.2)	4.6 (2.8-6.1)	**0.004**
	**MRC**	-	5 (4-5)	
	**HHD**	-	16.3 (8.4-21.8)	
**Trap**	**MUNIX**	198 (172.7-247)	139.5 (105-190.5)	**0.001**
	**MUSIX**	39.6 (37.8-41.5)	40.5 (38.1-43.2)	0.276
	**CMAP**	8.3 (7-9.4)	5.8 (4.6-7.2)	**0.001**
	**MRC**	-	5 (4-5-5)	
	**HHD**	-	16.3 (10.3-22.8)	

Parameters are given as median (25°-75° percentile). # Missing data in n=1 HS. Abbreviations: ALS = Amyotrophic lateral sclerosis; APB = abductor pollicis brevis; ADM = abductor digiti minimi; CMAP = compound muscle action potential; FDI = first dorsal interosseus; HHD = hand-held dynamometry HS = healthy subjects; MRC = Medical Research Council (scale); MUNIX = Motor Unit Number Index; MUSIX = Motor Unit Size Index; TA = tibialis anterior; Trap = trapezius. CMAP is given as mV, HHD is given as kilogram-force.

Table S2. Individual MUNIX parameters per muscle in ALS.

n	TA		APB		ADM		FDI		Trap	
	MUNIX	MUSIX	MUNIX	MUSIX	MUNIX	MUSIX	MUNIX	MUSIX	MUNIX	MUSIX
**1**	84,2	51,1	55,7	51	80,5	57,8	72,4	53,2	162	38,7
**2**	34,5	56,6	30,9	47,5	98,7	56,8	71,1	73,4	87,1	47,7
**3**	119	54,6	215	47,4	196	56,3	248	65,7	247	35,2
**4**	63,6	51,4	114	51,9	155	55,9	229	53,1	186	38,4
**5**	139	44,1	**2**	**250**	**2**	**250**	**2**	**250**	89,5	53
**6**	162	45,4	152	47,5	161	53,6	246	53,9	211	38,6
**7**	97,7	53	220	47,4	181	50,2	205	48,4	152	38,8
**8**	53,5	51,1	104	50,2	170	50,7	138	62,7	138	41,4
**9**	26,7	75,6	59,1	66	79,7	59,9	90,2	65,7	101	41,2
**10**	82,3	50,7	55,7	69,6	49,7	53,8	80,5	58,2	129	38,4
**11**	162	52,4	58,5	64,8	71,9	98	150	74,6	141	48,9
**12**	108	56,5	138	48,6	192	48,8	278	53	225	42,3
**13**	105	46,4	136	50,9	188	51,9	208	51,2	134	37,9
**14**	68,7	56,6	12,3	48,7	36,1	69,9	45,1	67,8	117	41,2
**15**	29,2	59	124	62	82,7	66,2	182	69,1	192	38
**16**	89,4	54,4	142	40,8	146	50,8	192	60,9	254	40
**17**	145	48,2	149	47,2	144	62,7	147	73,3	228	36
**18**	72,5	48,7	48	95,2	72,4	78,9	185	62,7	179	38
**19**	89,2	55,7	40,6	55,1	71,2	86,6	80,9	76,7	90,4	43,5
**20**	147	50	56,3	75,2	7,7	368	16,5	224	126	36,5
**21**	45,9	57,8	63,6	51,1	24,1	61,5	29,3	119	74,8	42,5
**22**	**2**	**250**	29,5	82,3	68,6	74,9	154	52,6	29,6	52,8
**23**	72,1	51,5	**2**	**250**	26,8	83,1	30,5	104	142	45,8
**24**	125	47,9	110	52	113	54	141	51,1	138	41,1

Abbreviations: ALS = Amyotrophic lateral sclerosis; APB = abductor pollicis brevis; ADM = abductor digiti minimi; FDI = first dorsal interosseus; MUNIX = Motor Unit Number Index; MUSIX = Motor Unit Size Index; TA = tibialis anterior; Trap = trapezius.

Correlation analysis

Correlations between MUNIX parameters and CMAP, MRC and HHD values per each muscle, adjusted for the effects of age, sex, and disease duration:

APB-MUNIX correlated with CMAP, MRC and HHD values (respectively: r=0.970, p<0.001; r=0.574, p=0.006; r=0.805, p<0.001), while APB-MUSIX correlated with MRC values (r=-0.898, p<0.001) but not with CMAP and HHD values (respectively: r=-0.428, p=0.053; r=-0.298, p=0.189).

ADM-MUNIX correlated with CMAP, MRC and HHD values (respectively: r=0.955, p<0.001; r=0.566, p=0.008; r=0.693, p<0.001), while ADM-MUSIX correlated with CMAP values (r=-0.530, p=0.013) but not with MRC and HHD values (respectively: r=-0.126, p=587; r=-0.326, p=0.150).

FDI-MUNIX correlated with CMAP, MRC and HHD values (respectively: r=0.958, p<0.001; r=0.546, p=0.010; r=0.727, p<0.001), while FDI-MUSIX correlated with CMAP and MRC values (respectively: r=-0.551, p=0.010; r=-0.461, p=0.036) but not with HHD values (r=-0.374, p=0.095).

TA-MUNIX correlated with CMAP, MRC and HHD values (respectively: r=0.986, p<0.001; r=0.573, p=0.007; r=0.450, p=0.041), while TA-MUSIX correlated with CMAP and MRC values (respectively: r=-0.444, p=0.044; r=-0.872, p<0.001) but not with HHD values (r=-0.329, p=0.145).

Trap-MUNIX correlated with CMAP, MRC and HHD values (respectively: r=0.975, p<0.001; r=0.505, p=0.019; r=0.595, p=0.004), while Trap-MUSIX correlated with CMAP values (r=-0-487, p=0.025) but not with MRC and HHD values (respectively: r=-0.254, p=0.267; r=-0.159, p=0.491).
